# Recent Trends in Active and Passive Immunotherapies of Alzheimer’s Disease

**DOI:** 10.3390/antib12020041

**Published:** 2023-06-19

**Authors:** Meshal Alshamrani

**Affiliations:** Department of Pharmaceutics, College of Pharmacy, Jazan University, Jazan 45142, Saudi Arabia; malshamrani@jazanu.edu.sa

**Keywords:** Alzheimer’s disease, amyloid β peptide, astrocytes, intravenous immunoglobulin, microglia, tau

## Abstract

In the elderly, a debilitating condition known as dementia, which is a major health concern, is caused by Alzheimer’s disease (AD). Despite promising advances by researchers, there is currently no way to completely cure this devastating disease. It is illustrated by the deposition of amyloid β-peptide (Aβ) plaques that are followed by neural dysfunction and cognitive decline. Responses against AD activate an immune system that contributes to and accelerates AD pathogenesis. Potential efforts in the field of pathogenesis have prompted researchers to explore novel therapies such as active and passive vaccines against Aβ proteins (Aβ immunotherapy), intravenous immunoglobulin, and tau immunotherapy, as well as targets that include microglia and several cytokines for the treatment of AD. Aims are now underway by experts to begin immunotherapies before the clinical manifestation, which is made possible by improving the sensitivity of biomarkers used for the diagnosis of AD to have better outcome measures. This review provides an overview of approved immunotherapeutic strategies for AD and those currently being investigated in clinical trials. We examine their mechanisms of action and discuss the potential perspectives and challenges associated with immunotherapies for AD.

## 1. Introduction

Alzheimer’s disease (AD) is an age-related neurodegenerative disorder. Clinically, AD is known for progressive memory impairment, deficits in cognitive abilities, and alterations in personality and behavioral traits [1]. This deterioration is more prominent in the hippocampal and temporal regions [2]. According to a 2017 Alzheimer’s Association report, approximately 5.5 million Americans are suffering from AD, and it is expected to increase to 13.8 million by mid-century in the US. Currently, the rate of AD development is one in every 66 s, which may reach 33 s by 2050, which will add one million new cases per year to the list. In the decade 2000–2014, the mortality rate of AD increased to 89%, while prostate cancer, heart disease, and stroke decreased to 9%, 14%, and 21%, respectively. In 2014, AD became the sixth-most deadly disease in the United States, causing 93,541 deaths. Its treatment is extremely expensive. Average AD health care and treatment costs of >$230 billion, which may increase for patients aged ≥65 years, were estimated to be $259 billion in 2017 [3]. Moreover, most U.S. nationals are getting to the age of 65 or more, which will increase the number of AD and dementia patients. It is projected to increase from 58 million in 2021 to 88 million in 2050, both in number and proportion [4].

AD is divided into two major categories: familial and sporadic. Familial Alzheimer’s disease (FAD) is a rare early-onset form of Alzheimer’s disease caused by mutations in three major genes: amyloid precursor protein (APP), presenilin 1, and presenilin 2. On the other hand, sporadic Alzheimer’s disease (SAD) is the most common cause of dementia and accounts for more than 90% of Alzheimer’s cases. SAD tends to strike people without a family history of the disease and occurs late in life, after the age of 65. The clinical features of FAD and SAD are similar, and they do not differ in the incidence of risk factors for dementia or MRI or PET features. However, FAD has an early onset before 65 years of age, whereas SAD has a mean age of onset over 70 years. Understanding the genetic and clinical differences between FAD and SAD is essential for developing effective treatments and management strategies for this devastating disease [5,6]. Patients with mild cognitive impairment are at a higher risk of developing AD and are sometimes considered to be in an early stage of AD [7].

Presently, the actual cause of AD is unknown, so no efficient disease-modifying therapies are available. Even though many pharmacological strategies are used to delay the progression of cognitive impairment and memory loss, to fight this disease, it is essential to discover novel therapeutic targets [8,9]. Thus, this improved immune-related insight will deliver attractive biomarkers and novel therapeutic targets for the diagnosis and monitoring of AD [10,11,12]. Aβ immunotherapy results in plaque elimination and functional benefit. Numerous successive clinical trials that include both active (AN1792) and passive immunization (e.g., Bapineuzumab and Solanezumab) have been verified by autopsy neuropathology and in vivo imaging, which shows that Aβ from the human brain can be eliminated. However, until now, the evidence for unequivocal cognitive benefits has been slightly unsatisfactory [8]. This review provides an overview of approved immunotherapeutic strategies for AD and those currently being investigated in clinical trials. We examine their mechanisms of action and discuss the potential perspectives and challenges associated with immunotherapies for AD.

## 2. Etiopathophysiology of AD

**Figure 1:**
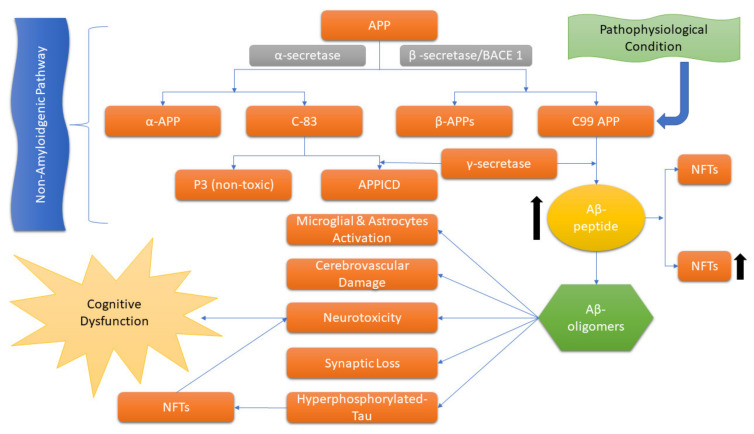
Schematic representation of the amyloidogenic APP pathway. APP: amyloid precursor protein; APICD: intracellular domain of the amyloid precursor protein; NFTs: neurofibrillary tangles.

Usually, AD is illustrated by the extracellular accumulation of β-amyloid (Aβ) and intracellular neurofibrillary tangles (NFTs) formed by hyperphosphorylation of the tau protein that leads to synaptic dysfunction and neuronal death [5,13]. The “amyloid cascade hypothesis”, a longstanding hypothesis that has a major role in AD, is proposed by Hardy and Higgins [14], which suggests that the APP by proteolytic cleavage results in the accumulation of insoluble Aβ fragments that is the key stimulus in driving AD pathology. While the imbalanced Aβ production and clearance result in further disease progression, including NFT formation (Figure 1) [15].

An amyloid plaque contains Aβ 1–42 peptide, i.e., a derivative of membrane-stacked APP, which is the main component that gets organized into dense fibrils and intermingles with non-fibrillar peptide. Plaques also hold deteriorating axons and dendrites, and these are encircled by reactive astrocytes and some microglia [16]. NFTs comprise tau proteins associated with microtubules that are generally expressed in axons. However, in AD pathogenesis, hyperphosphorylation of tau leads to the aggregation of abnormal filaments in the cell body, thus impairing the regular activity of tau in the polymerization and stabilization of tubulin. This hyperphosphorylation of tau is performed by mitogen-activated protein kinases (MAPK) due to the overexpression of MAPK/ERKs in an AD brain [16].

The inflammatory response in AD pathology is chiefly driven by microglia and intensifies with disease progression, which indicates the role of the immune system in AD; however, the significance of inflammation in AD pathogenesis is well appreciated and considered to contribute to and exacerbate AD [10,17].

AD is characterized by two distinct pathological features: extracellular deposits of beta-amyloid in neuritic plaques and intracellular neurofibrillary tangles composed of paired helical filaments. These abnormalities result in synaptic and neuronal loss, leading to noticeable brain atrophy, particularly in the mesial temporal lobe [18,19].

The precise mechanisms by which beta-amyloid and neurofibrillary tangles cause damage in AD are not yet fully understood, giving rise to several proposed theories. The amyloid hypothesis suggests that the progressive buildup of beta-amyloid triggers a complex cascade of events that ultimately culminate in neuronal cell death, synapse loss, and neurotransmitter deficits, contributing to the clinical symptoms of dementia [20,21].

Inflammation and immune responses have emerged as significant contributors to AD pathology, potentially representing a third core pathological feature. Disturbances in glucose metabolism have also been implicated in the development of AD [22,23].

AD shares similarities with prion diseases, as beta-amyloid and tau proteins exhibit prion-like properties. These proteins can self-replicate and contribute to disease progression by inducing misfolding in their normal counterparts, leading to abnormal accumulation and subsequent brain damage. Further research is necessary to fully comprehend the intricacies of these mechanisms and their implications for AD pathogenesis [24,25,26,27].

## 3. Involvement of the Immune System and Inflammation in AD

Recent bioinformatics, genetic, and preclinical data highlight that neuroinflammation mediated by immune activation exacerbates and contributes to AD pathogenesis [28]. Immense anomalous interactions have been found among Aβ, neurons, astrocytes, and microglia of the central nervous system (CNS) in the proteinopathy AD of the elderly that employ a malicious cycle in AD immune-neuropathology [29]. These multicellular interactions occur due to the accumulation of cytotoxic proteins (insoluble NFTs and Aβ plaques) in definite regions of the brain. Insoluble NFTs and Aβ plaques are crucial factors in AD pathogenesis by forming senile plaques in the brain [30,31]. Due to proteinopathy, these neuronal cells initiate the recruitment, immigration, and aggregation of astrocytes and microglia near affected neurons. Microglia are the mononuclear phagocytes that normally act as the protective cells of the CNS, while astrocytes are the supporters of neuron cells that supply nutrients and maintain pH by accumulating at synapses. Damaged neurons initially attract microglia by secreting fractalkine (CX3CL1), which acts on the microglial receptor CX3CR1, as well as an astrocyte-released glial-derived neurotrophic factor (GDNF), which interacts with the GDNF receptor [32,33,34,35].

Neuronal oligomeric Aβ42 in neurodegenerative lesions directly attracts microglia and, to a lesser extent, astrocytes through a wide range of receptors, most prominently TLRs 4, TRLs 6, and CD36 [34,36,37]. Astrocytes are also attracted to the neurodegenerative lesion by CC chemokine ligand 2 (CCL2) and monocyte chemoattractant protein-1 secreted by activated astrocytes, neurons, and particularly activated microglia [33]. Blood monocytes are also drawn to such lesions through CCL2 and CC chemokine receptor (CCR2) interactions, which then differentiate to form more potent macrophages than senescent microglia and exacerbate the ongoing pathogenesis by promoting inflammation [38,39]. Increased accumulation of neuronal cells (astrocytes, microglia) around the damaged neuron (induced directly or indirectly (through cytokines) by neurotoxic proteins) leads to multicellular interactions that cause further activation of cells and elicit more pathogenic alterations. IL-34 secretion by neurons and sometimes by T cells and dendritic cells increases microglial survival, proliferation, and function (cytokine production) by acting on the colony-stimulating factor-1 receptor [34,40].

Microglial-derived cytokines work in combinations (TNF-α and IFN-r or IL-1β and IFN-r) to stimulate astrocyte proliferation and enhance the production of the precursors of the amyloidogenic pathway that include APP and β-site APP cleaving enzyme 1 (BACE-1) that are delivered efficiently by astrocyte-derived exosomes (ADE) to neurons and stimulate abnormally elevated Aβ42 production [41,42,43]. At that stage, activated cells are fully equipped to attain peak neuronal destruction through diverse mechanisms such as complement-mediated lysis, cytokine-induced damage, pruning of neurons by phagocytosis, and mitochondrial transfer [44].

In chronic neurodegenerative lesions, neuronal, astrocytic, and microglial effects undergo alteration, resulting in increased product secretions and prolonged destructive effects on other cell types. In AD, increased secretion of cytokines by inflammatory microglia further promotes astrocyte production and activation, which in turn increases the secretion of chemokines (CXCL10, CCL3, and CCL5) that further activate microglia [44]. GDNF levels, either from activated astrocytes or inflammatory-type microglia that normally enhance synaptic function, neuronal survival, or plasticity, may be elevated or diminished in AD depending upon the disease stage [43,45,46].

Activated astrocytes (particularly under ischemic conditions) are capable of transferring mitochondria into the neurons to increase or decrease neuronal survival, but this role is still under analysis for AD [47]. Activated microglia that become rich in Aβ plaques enhance the inflammatory response by stimulating NFκB (a nuclear factor) and by regulating the MAPK pathway and extracellular signal-regulated kinase that is involved in cytokines and chemokines production [48,49]. These molecules, in conjugation with free radicals and complement components, intensify neuronal dysfunction, which eventually leads to death [50].

Inflammatory responses can also be driven by both CNS intrinsic and extrinsic factors (systemic influence). Intrinsic conditions (e.g., locus coeruleus degeneration, traumatic brain injury) and extrinsic conditions (type 2 diabetes, obesity, and systemic inflammation due to chronic disorders) are involved in neuroinflammation and microglia activation that facilitate AD pathogenesis [51,52,53,54,55].

Recently, the relationship between genes of innate immunity and AD pathogenesis has been found in sporadic AD by genome-wide association studies, as shown by AD-associated mutations in myeloid genes that encode triggering receptors expressed on myeloid cells 2, CD33, a surface antigen, and complement receptor 1 (CR1) [56,57,58,59].

## 4. Diagnoses

A well-known pathological hallmark of AD is Aβ peptides and NFTs comprised of phosphorylated tau (p-tau) [60]. An analysis by Rajan et al. has revealed that cognition and memory impairment initiate about 18 years before clinical diagnoses of AD [61]. Thus, AD cannot be diagnosed at an early stage, and when the symptoms appear and AD is clinically diagnosed, then neurodegeneration has already reached significantly advanced stages with marked neural and synaptic dysfunction [62]. Late clinical diagnosis is a consequence of nonspecific diagnostic tests. Early AD diagnosis is often subjective and determined by GPs (usually neurologists, psychiatrists, and geriatricians) and depends upon their experience [63].

The rising incidence of AD and neurodegenerative pathogenesis demands the urgent development of reliable biomarkers that can be identified at the preclinical stage for precise diagnosis and efficient monitoring of the disease [64]. Recently, intensified efforts have been made to develop such AD biomarkers that can be detected in cerebrospinal fluid (CSF), blood, and brain imaging [65,66]. Here, CSF biomarkers are the most important as they depict diseased-brain neuropathology [64]. For AD, the major CSF biomarkers are Aβ42, p-tau, and total-tau (t-tau). Apart from CSF biomarkers, plasma t-tau and neurofilament light protein (NFL) are also associated with AD [67]. CSF protein profiles provide a better understanding of brain pathological changes. AD and mild cognitive impairment patients have an elevated level of CSF p-tau (Thr 181) and t-tau, while the declined level of CSF Aβ42 reflects plaque pathology [68,69,70]. In the CSF, FAD mutation carriers (APP, Presenilin-1) also have elevated total and p-tau (Thr 181) levels, which is a sensitive indicator of pre-symptomatic AD [71].

Besides biomarkers, neuroimaging approaches provide a noninvasive and precise assessment of neuronal function [72]. The most common efficient techniques include functional magnetic resonance imaging (fMRI), magneto-encephalography (MEG), electroencephalography (EEG), single-photon emission computed tomography (SPECT), positron emission tomography (PET), and functional near-infrared spectroscopy (fNIRS) [73]. Computerized diagnostic tools examine the cerebral neurodegeneration pattern from the images to diagnose AD. MRI and PET techniques quantify brain changes and metrics such as white and gray matter, cortical thickness, and hippocampus shape and volume for AD progression. Moreover, 18-Fluoro-DeoxyGlucose PET (FDG-PET) identifies the glucose metabolic rate at cerebral regions that assist in AD diagnosis [72].

## 5. Current Treatments for AD

Currently, no proper medicinal treatment is available for AD that slows or inhibits neuronal destruction; as a consequence, AD symptoms appear, thus making the disease fatal. Hence, the need for efficient treatments has increased greatly to monitor AD progression [74]. In the last 30 years, significant developments have been made to understand the genetics, neuropathology, and biochemistry of AD [9,75]. Unfortunately, only six drugs are approved by the Food and Drug Administration (FDA), USA, for the symptomatic treatment of AD by increasing the concentration of neurotransmitters in the AD brain. These drugs vary in their effectiveness from individual to individual and have a limited duration [3,76].

Memantine (a NMDA receptor antagonist) and acetylcholinesterase inhibitors (AChEIs) are currently used for the management of AD symptoms, but these drugs cannot inhibit disease progression due to their limited effectiveness [77]. Several disease-modifying drugs were found unsuccessful in clinical trials as they exhibit mechanism-based side effects. These drugs are mainly Aβ-targeted and comprise γ-secretase or β-secretase inhibitors, which prohibit Aβ production [75]. In the 2002–2012 decade, 244 AD drugs registered by the clinical trials government were tested in clinical trials. Disappointingly, only one out of 244 drugs got approval from the FDA after completing the clinical trials [3].

After the confirmation of inflammation-mediated AD pathogenesis, anti-inflammatory drugs, mainly nonsteroidal anti-inflammatory drugs (NSAIDs), that were thought to protect against AD are tested as a therapeutic option in a clinical trial. However, despite these promises, not even a single drug is involved in slowing the progression of cognitive dysfunction in mild to moderate AD patients. Therefore, NSAIDs as well as other approaches targeting metals, oxidative damage, etc. are no longer used as a viable treatment for AD [78,79]. Various factors contribute to this difficulty in the development of effective AD treatment, including the high costs of drug development, the fact that only specialized small drug molecules can move across the blood-brain barrier (a protective layer) of the brain, and the lengthy observation period required to investigate the effectiveness of treatment [3].

Recently, the discovery of AD biomarkers and advancements in biomolecular mechanism identification have directed that novel therapies modify early-phase pathogenic mechanisms. As Aβ deposition is the key incident in AD pathogenesis, numerous therapeutic approaches have been developed that are involved in Aβ clearance and impede Aβ production or aggregation. Among these strategies, immunotherapy is the most researched therapeutic approach [80]. Srivastava et al. suggested combined therapies for the treatment of AD. Multi-targeted drugs can be used, but no potential drug has been developed beyond phase II/III trials. Still, problems remain with difficulties in crossing the blood-brain barrier, a long serum half-life, and low bioavailability. Researchers are now interested in miRNA replacement therapy for AD treatment with restricted interactions [1].

## 6. Immunotherapy and Its Types

A promising approach for combating AD is amyloid immunotherapy, which includes various anti-Aβ strategies such as the production of anti-Aβ antibodies that are being fashioned actively (vaccines) or passively to prevent Aβ peptide aggregates and lower their production [81,82]. To excite the immune system in the host that affects the production of anti-Aβ antibodies, Aβ active immunization involves the processing of synthetic Aβ peptide conjugated to a carrier protein, while in passive immunotherapy, there is no requirement to hook up the immune system; instead, Aβ-specific antibodies are directly injected into the host [80,82].

## 7. Preclinical Immunotherapy Trials in Animal Models

APP is the contributory gene liable for the protein, taking account of the Aβ peptide. In 1980, Aβ deposits ensured sequencing, and APP was cloned by Glenner. To dissipate Aβ deposits and prevent monomers from aggregating, experiments were performed in vitro that distort the Aβ peptides [83]. Preclinical immunotherapy for AD in transgenic mice (Tg) showed the ability to reduce or abolish Aβ pathology by overexpressing a mutant Aβ precursor protein (AβPP) with Aβ42; this was first reported in 1999 [84]. After this trial, no noticeable lethal activity was spotted. In later studies, similar fallouts were set and completed by using Aβ42 with the addition of alum adjuvants, which not only precluded Ab plaque but also advanced cognitive functions [85,86]. Vaccinating the mouse before acquiring pathology abridged levels of cerebral amyloid that were already present in the transgenic mouse and produced high serum antibody titers [80]. This observation that memory deficits are treated by exploiting antibodies for the Aβ peptide has been extensively practiced in these models, demonstrating that even in some cases, an action (instantly) after a short period of treatment is spotted [87]. Other than mice, Beagle dogs that had built up a diffused type of Aβ deposits that also showed memory aberration with age when treated for two years responded to clearance of diffused deposits but did not fix the memory impairments [88].

## 8. Contributory Factors behind AD and Mechanisms of Their Clearance

AD is rooted mainly because of three reasons: Aβ deposits, α-synuclein (α-syn) accumulation in various brain sections, and the dissemination of tau in a prion-like fashion [89,90,91]. Lethality in AD is also yielded by oligomers’ presence in the brain, imparting deleterious effects such as damage to synaptic function, disturbances in autophagy, and gene transcription [92,93,94]. Recently, it has been stated that there is an intermingling of protein accretion [95].

**Figure 2:**
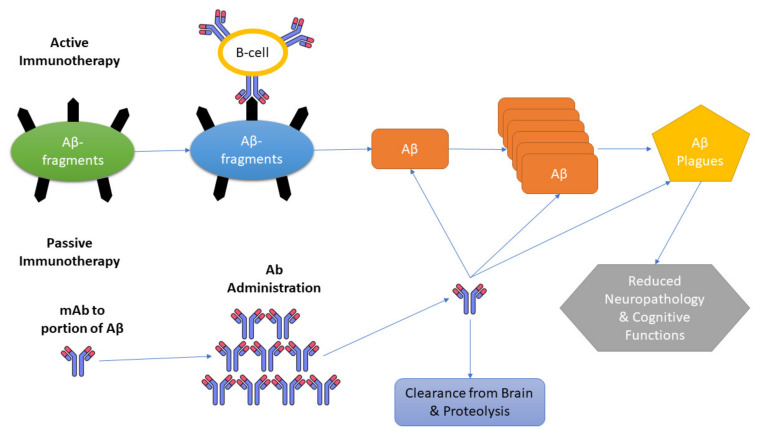
Mechanisms of Aβ clearance by active and passive immunotherapy. Aβ: β-amyloid; mAb: monoclonal antibody.

Three mechanisms have been presented as immunotherapies for AD. Initiation of microglia and macrophages; secondary structure modification of Aβ monomer; peripheral sink hypothesis, which states that by upsetting the constancy of Aβ among the plasma and CNS, there is an escalation of the outflow of Aβ from the brain (Figure 2) [96].

### 8.1. Initiation of Microglia and Macrophages

In the first proposed mechanism, the opsonization technique is implicated, in which the pathogen is tagged, leading to macrophage phagocytosis and complement activation. The basic principle of this approach presumes that an adequate amount of antibody leads into the brain to regulate inflammation by activating clearance through microglia. This response is possible when these antibodies fasten to the amyloid, and after systemic administration of anti-amyloid antibodies, recovery from amyloid deposits has been demonstrated [97,98,99].

### 8.2. Peripheral Sink Hypothesis

This hypothesis involves the creation of a “peripheral sink”, which leads to the withdrawal of Aβ from the brain via anti-Aβ antibodies (Aβ-specific IgG) [100,101]. The high affinity for Aβ of many antibodies does considerably elevate circulating concentrations, and many mechanisms appear there by which entry into the CNS is made by circulating Aβ; this reduces free Aβ in the brain [102].

### 8.3. Aβ Oligomers Neutralization by Antibodies

The presence of soluble aggregates called “oligomers” in elevated amounts accounts for AD even if no genetic abnormality is seen in the patients [103]. A third approach involves secondary structure modification of Aβ monomers by using the catalytic characteristics of antibodies. This confirms that oligomers do not amass. Mechanism suggests that the disbanding of amyloid composites occurs through a straightforward effect on Aβ because here Aβ antibodies act as chaperones [83]. For the blockage of Aβ fibril formation in vitro, the required stoichiometry of antibodies is as low as 1:10. These outcomes were further extended, which concluded that atomic force microscopy (AFM) indicates that antibodies in a ratio of 1:1000 are needed to obstruct oligomers [104].

## 9. Active Immunization (Vaccine) Clinical Trials

Further, immunohistochemical assays made their way to clinical trials in humans so that immune interventions in humans could also be performed after observing results in mice, which revealed that immunization antibodies hostile to Aβ can also be beneficial in human AD eradication by acting on brain segments [86,105,106].

No toxicity was seen in the immunized mice after preclinical trials. In April 2000, firstly, the trial was put into practice that applied the AN1792 (beta-amyloid [Abeta]1-42) vaccine that activated the immune system because of the incidence of adjuvant (Qs21) and pre-amassed [107]. In the U.K., the above-described study accomplished and performed accounts for 80 patients having an AD at a stage that is considered to be weak to medium [108]. Phase 1 is conducted, and its chief objective is to check the arousal of immune activity upon entry of the vaccine. After testing multiple times, results demonstrated that more than 50% of patients could acquire an increased opposing response to Aβ. To further escalate the progress of the immune system, an emulsifier, polysorbate 80, is added, which benefits it more [109].

The execution of Phase II in October involved a total of 372 patients, of whom 300 were given a higher dose of vaccine along with emulsifiers in a 4:1 ratio. On receiving such a high dose, symptoms of aseptic meningoencephalitis were exhibited by 6% of immunized patients, and early in January 2002, the trial was completed [107,110,111]. The extent to which symptoms continued is 5–168 days and includes headache, lethargy, and confusion. Likewise, the data from the animal model and the analysis of a postmortem-evidenced deduction of Aβ peptide masses endorsed the efficacy of the approach [111,112,113,114,115]. Plaques have a moth-eaten appearance, or, in other words, naked, dense interiors. Histopathology also revealed that these plaques are associated with microglia and involve phagocytosis for clearance, and it also revealed that tau-related pathology is not targeted [113,114]. The toxic comeback was marked when further trials were conducted in vitro, in which peripheral mononuclear cells from patients were examined upon triggering by the Ab peptide. Cytokines are produced and quantified by ELISA, which shows the production of interleukins and interferon-gamma by the patient’s cells; this is generated by CD4^+^ receptors of T-helper. Stated that a more worthy result is obtained by applying a high amount of antibody solutions; this information is obtained by following the Zurich cohort results [109]. With such a remarkable fall in plaques, the benefits obtained in cognitive function are negligible, while the result in pathology is remarkable [110]. Various assessments of active immunotherapy vaccinations are still proceeding worldwide, especially in the USA [116,117].

## 10. Active Immunotherapy Antibodies

The first active immunotherapy approach used was AN1792, with promising results of reduced functional decline, but its use was stopped clinically due to meningoencephalitis in the immunized diseased persons [110]. This was actually due to the activation of T helper (Th1) cells of the immune system, which in turn stimulate proliferative function.

CAD106 has recently devised an antibody-right formulation against Aβ deposits where it impedes the Aβ peptides [118]. The Phase 1 assessment of CAD106 gives insight into the view that many of the patients produced anti-Aβ IgM immunoglobin and less produced IgG against Aβ [119,120].

ACC-001 is an enduring active Phase II trial that involves a small Aβ fragment and active saponin adjuvant QS-21 with a carrier protein [121]. It persuades such a proficient action that is nontoxic, and its study is still being evaluated at present.

AFFITOPE AD02, recently renamed AD04, targets T-cell activation to evade the adverse effects of AN1792. Its amino-terminal B cell epitope greatly lowers the Aβ plaques, and it is clinically proven [122]. Table 1 represents the active immunotherapy clinical trials: advancements in promising treatments.

### 10.1. Tau Immunotherapy

Over time, the efforts to develop tau immunotherapy and tau-related neurodegenerative disorders have increased immensely in the past couple of years, maybe to a limited extent, because of the disappointment of Aβ immunotherapy in inverting cognitive deficiencies in moderate to severe AD [123]. Currently, tau-directed treatments are not considered as advanced as other therapies developed for modifying AD. The accomplishment of a tau-directed treatment is difficult because tau protein is intracellular [124]. However, various examinations have demonstrated the uptake of antibodies by neurons. Furthermore, the cellular stress and inflammatory changes caused by tau pathology may encourage the uptake of antibodies, principally into damaged neurons, conceivably lessening undesirable symptoms. Antibodies may also inhibit the extracellular spread of tau pathology [125,126,127].

Up until now, approximately eight preclinical reports have been publicized concerning the useful impacts of active and passive therapy targeted to tau phospho-epitopes or tau aggregates in the tau Tg mouse model [123]. In P301S tau transgenic mice, monoclonal antibodies showed diminished microglial activation, blocked the progression of tau seeding activity identified in cerebrum lysates, and enhanced cognitive abilities [128]. Moreover, tau immunotherapy has been found to prevent extreme cognitive debilitation through the extensive removal of abnormal tau [129]. In another investigation, tau expression is suppressed in transgenic P301L mice, which demonstrates improved memory, although NFTs remain [130]. Thus, preclinical studies in different mouse models recommend that tau immunotherapies with phosphorylated peptides decrease tau phosphorylation and NFT load when treatment is initiated earlier or around the beginning of NFT pathology, demonstrating that the clearance of early pathological aggregates may have a therapeutic advantage [131,132].

However, the absence of lucidity in regards to which conformer of tau ought to be directed in this manner remains an issue [133]. Overall, all these investigations bolster the viability of targeting pathological tau in AD patients.

### 10.2. ACI-35

ACI-35 (AC Immune, Lausanne, Switzerland) is a liposomal vaccine that contains a synthetic peptide analog (16 amino acids) of the human tau protein sequence from 393 to 408, with phosphorylation at S396 and S404 residues utilizing a similar technique as ACI-24. In tau P301L transgenic mice and wildtype mice, ACI-35 evoked fast and robust polyclonal antibody reactions specific to p-tau [134]. The tolerability of the vaccine was also shown by upgraded clinical attributes and the absence of brain inflammation. This data indicates that ACI-35 could be a safe and viable treatment for AD patients.

### 10.3. AADvac1

It is a KLH-conjugated tau peptide that is administered with Alhydrogel (aluminum hydroxide adjuvant) and is the first clinically developed vaccine that specifically targets misfolded tau proteins. Transgenic mice and rat vaccination by AADvac1 active and passive therapy decreased NF degeneration and mortality as well as improved neurobehavioral deficiencies [135]. The AADvac1 active vaccine is still under observation in a randomized, Phase 1, 3-month clinical trial to assess its efficacy, safety, and tolerability in mild-to-moderate AD patients (ClinicalTrials.gov NCT01850238).

**Table 1:** Active immunotherapy clinical trials.

Aβ Active Immunotherapy Clinical Trials								
Drug	Sponsor	Vaccine Type	Target (Aβ/Tau)	Trial Phase and Status	Immunology	Positive Outcomes	Negative Outcomes	References
AN-1792	ELAN (Dublin, Ireland)	Anti-Aβ vaccine (Aβ 1–42 with Qs21 as adjuvant).	Aβ N-terminus	II Halted, no improvement (NCT00021723)	Induction of anti-Aβ titers by B and T-cell activation.	↓↓CSF tau and no change in CSF Aβ 42 level.	~6% of cases developed Meningoencephalitis and cerebral microhemorrhage	[136]
CAD-106	Novartis (Basel, Switzerland)	Anti-Aβ vaccine Aβ 1–6 peptide along with QB coat protein of bacteriophage.	Aβ N-terminus (AB1-6)	II Ongoing (NCT00956410, NCT01023685, NCT00795418, NCT01097096, NCT00733863, NCT00411580)	Induction of anti-Aβ titers without T-cell activation.	Safe and well-tolerated, ↑↑Total serum Aβ, ↓↓and free Aβ in plasma while CSF t-tau, p-tau, and Aβ-40 and 42 remain unchanged.	The occurrence of ARIA in a few cases.	[137,138,139]
ACI-24	AC immune (Lausanne, Switzerland)	Tetra-palmitoylated peptide (Aβ 1–15) re-formed in liposome.	B sheet conformation of Aβ	I/II Ongoing (NCT02738450)	The non-inflammatory response of Th2 helper cells against Aβ.	↓↓ insoluble Aβ40 and 42 and soluble Aβ42.	No significant adverse effects.	[117,137,138]
ACC-001	Pfizer (New York, NY, USA)/Janssen (Titusville, NJ, USA)	Anti-Aβ vaccine Aβ 1–7/non-toxic diphtheria toxin (CRM197)/Qs21 adjuvant.	Aβ 1–7	II (Completed) Additional Phase II is ongoing(NCT01284387, NCT00955409, NCT01227564, NCT00960531, NCT01238991, NCT00752232, NCT00959192, NCT00498602, NCT00479557)	Induces antibody’s response against Aβ.	Safe and well-tolerated, ↑↑plasma Aβ40, ↓↓CSF p-tau slightly, while other CSF biomarkers remain unchanged.	Local injection reactions and headaches; ARIA-E occurs in few cases.	[140]
AD02	GlaxoSmithKline (Brentford, UK)/AFFiRiS (Vienna, Austria)	Aβ 1–6 mimetic/keyhole limpet hemocyanin/aluminum adjuvant.	Mimotope of Aβ N-terminal	II Ongoing (NCT01093664, NCT01117818, NCT02008513, NCT00633841, NCT00711321, NCT01357629, NCT01614132, NCT00003453, NCT00996008)	Stimulate the immune system to make antibodies against Aβ.	Safe; no detailed outcomes.	The non-endogenous nature of drugs avoids the development of tolerance.	[138]
V-950	Merck and Co. (Kenilworth, NJ, USA)	Aβ amino-terminal conjugated to ISCO-MATRIX.	Aβ	I (Discontinued) (NCT00464334)	Production of anti-Aβ antibodies.	Results unpublished.	AE’s rate is high. Mostly Fatigue, nausea, anemia diarrhea, while in a few cases arrhythmia, dysphagia.	[138,141]
Tau Active Immunotherapy								
AADvac1	Axon Neuroscience (Bratislava, Slovakia)	Anti-tau vaccine	Tau derived peptide (294–305 aa)	I (NCT02031198, NCT01850238, NCT02579252, NCT03174886)	Antibodies are directed against p-tau and promote tau clearance.	Safe; ↓↓tau aggregates. Improved cognition.	No significant adverse effects.	[135,138]
ACI-35	AC immune (Lausanne, Switzerland)	Anti-tau vaccine	Tau derived peptide (294–305 a.a)	I (NCT04445831)	Stimulate immune system B and T-cell response. Antibodies are directed against p-tau and promote tau clearance.	↓↓soluble and insoluble tau.	No significant adverse effects.	[138,142]

↑↑ (increase), ↓↓ (decrease).

## 11. Passive Immunotherapy

An effective approach that implies the injection of purified, epitope-specific antibodies to target the Aβ plaques in Alzheimer’s is passive immunization. The disadvantages of passive immunity, especially in the case of chronic disease, are the need for repeated injections, the selection of proper antigen targets, high costs, the risk of hemorrhages, and the induction of an immune response to the injected antibodies [86]. Several preclinical studies were initiated to exploit the ability of passive immunization, which included the treatment of mouse models of AD with Aβ42 immunotherapy. Results concluded were supportive, as a decline in the Aβ42 load was perceived, along with the finding that cognitive function is recovering [143]. In this study, adverse effects and cerebral microhemorrhages were also noticed [144,145]. To diminish microhemorrhages, modification of induced antibodies is accomplished with complement proteins and with Fc-g receptors, though with average competence, along with Aβ deposit reduction. Interestingly, there are several mechanisms to strap up the action of passive immunization by which AD pathology can be facilitated [86,146].

### 11.1. Antibody Bapineuzumab First-Generation Anti-Fibrillar Forms of Aβ

Currently, advances in passive immunotherapy are ongoing, and several trials are under study. Phase III trials in this respect comprise Bapineuzumab and Solanezumab, but when tried clinically, results were not satisfactory [147,148,149]. A humanized depiction targeting Aβ 1–5 deposits is an anti-Aβ monoclonal antibody, which is said to be Bapineuzumab. The fall in levels of CSF Aβ is significantly noticed in the results of Phase II clinical trials of Bapineuzumab; in addition to that impact, CSF p-tau and t-tau levels also showed a decrease [150,151,152]. To make advancements and seek therapeutic effects underlining safety, several large Phase II and III trials were conducted. In comparison to nanocarriers, the conclusions of Phase II trials that evoked different effects in ApoE4 carriers urged separate Phase III trials. A view by positron emission tomography (PET) in ApoE4 verified that Bapineuzumab has positive effects on brain amyloid with Pittsburg compound B, whereas there are no such impacts in the case of nanoparticles [153]. In these trials, noteworthy obstacles were related to amyloid-related imaging abnormalities (ARIA), parenchymal edema (ARIA-E), as well as intracerebral microhemorrhages (ARIA-H) [147,153]. There are intensified signals on MRI pulse sequences, probably due to leakage of the BBB in ARIA-E. Their reduction attempts were carried out by processing lower doses for apolipoprotein E e4 carriers, but still, several cases were diagnosed, so after all negative clinical outcomes, bapineuzumab has been terminated [154].

### 11.2. Antibody Solanezumab First-Generation against Soluble Monomeric Forms of Aβ

It is also the first generation, but it is a humanized IgG1 antibody that recognizes the middle sequence in between Aβ 16–24. Different properties of solanezumab and bapineuzumab are due to their diversified nature of binding with epitopes; Solanezumab recognizes a central domain epitope, whereas bapineuzumab targets aggregated forms of Aβ42 in the brain. Such a difference in reports lessened the adverse effects of Solanezumab as compared to Bapineuzumab [153,155]. In Expedition 1 and Expedition 2, patients with moderate AD were administered in two successive large Phase III trials for 80 weeks, and no adverse effects were reported. This indicated good safety trials, but cognitive function was not improved [148]. Later on, studies focusing only on mild AD patients accounted for a decline in cognitive function (33%) [156]. Following these results, a large-scale clinical trial (Expedition 3) on mild AD patients was prompted, but it was unsuccessful and showed no cognitive benefits. However, other trials are still ongoing with mild AD patients (Expedition PRO), as depicted on PET imaging by the presence of positive plaques [157,158].

Several other second-generation antibodies like AAB-003 (derived from bapineuzumab, Janssen/Pfizer) and GSK933776 that aid in minimizing inflammation and conformational antibodies against Aβ42 have been proposed and listed in the table that is also being applied clinically (Table 2).

## 12. Intravenous Immunoglobulin (IVIG) Immunotherapy

IVIG immunotherapy is an alternative approach to passive immunotherapy in which intravenous immunoglobulin (IVIG) is administered, which is a polyclonal antibody mixture obtained from the blood plasma of thousands of young and healthy volunteers [177]. It is an FDA-approved drug already being used for the treatment of various cancers, immunodeficiency syndromes, and neurological and inflammatory disorders [178]. An investigation concentrating on the potential treatment of AD started in 2002. It has been demonstrated that IVIG exhibits a low affinity for monomeric Ab proteins and a strong affinity for Ab fibrils and neurotoxic oligomers [179]. Dodel et al. described that the monthly administration of IVIG to five AD patients reduced CSF Aß, increased serum Aß, and enhanced cognitive functions [180].

Also, IVIG showed inflammatory and potent immune-modulating effects, vital for the potential treatment of AD. Preliminary clinical investigations revealed decreased cognitive decline. Phase II and III clinical trials have proceeded with IVIG administration in AD patients [181]. Generally, three Phases II/III clinical trials: Gammagard, Gamunex, and Octagam 10% were organized on IVIG therapy. A 10% IVIG Gammagard was tested in Phase III trials conducted by Baxter Healthcare Corporation in November 2012 in the US, enrolling 390 patients with mild to moderate AD for almost 18 months [86]. Unfortunately, the failure of positive outcomes leads to the termination of the IVIG program for AD (Table 3) (NCT00818662) [97,182,183].

Presently, games (one of the IVIG products) are actively tested in Phase II/III trials with the enrollment of 350 mild to moderate AD patients (NCT01561053). Recently, Octagam IVIG 10% has been observed in two Phase II trials. At first, a placebo-controlled, multicenter Phase II trial was conducted at five sites in Germany and seven in the USA [186]. This trial lost its preliminary endpoint of alteration in plasma Aβ levels and was found negative for most of its secondary biomarker results. While a second, single-centered Phase II trial reported possible effects on brain atrophy as seen by MRI and the Clinical Dementia Rating Sum of Box. The study is still dynamic, but it is not selecting new patients [187].

Generally, IVIG treatment was safe and well tolerated by the patients, even with multiple successive doses. Patients receiving regular infusions of IVIg have markedly decreased their risk of developing dementia [181,188]. Some positive outcomes have been observed in subgroups, particularly among APOE-e4 bearers and moderate AD patients. Due to the promising results of the initial studies, further trials, including IVIG, are being arranged, and currently enrollment is ongoing for more Phase III clinical trials (https://clinicaltrials.gov NCT01561053, accessed on 29 March 2023). It also demonstrates anti-inflammatory activity that dramatically increases circulating IgG and results in the regulation of various immune processes by a feedback mechanism until the level returns to normal. Therefore, IVIG can be suggested as an alternative treatment for AD.

## 13. Future Research and Limitations

Immunotherapy for AD remains a complex and unresolved area of research, necessitating further investigation into the mechanisms of antibody action and factors governing CNS antibody exposure. Passive immunotherapies offer a relatively safe option with enhanced target engagement, although their high cost makes them less viable for long-term public health solutions. Alternatively, the development of effective and safe vaccines holds promise as a cost-effective strategy for addressing the AD epidemic. Successful advancements in AD immunotherapy may revolutionize the treatment landscape for CNS disorders, extending the application of antibodies and vaccines to neurodegenerative, neurological, and psychiatric conditions.

Significant progress has been made in the non-invasive identification of AD, negating the need for autopsies. Positron emission tomography (PET) using amyloid binding ligands, including Pittsburgh compound B, allows for accurate differentiation of Alzheimer’s cases from other disorders. Ongoing research on ligands labeled with longer-lived isotopes, such as [18F], expands the possibilities of PET scans by eliminating the requirement for specialized facilities. Studies have demonstrated a reasonable correlation between PET ligand signals and amyloid deposition in the brain. Remarkably, some cognitively normal individuals exhibit positive PET amyloid ligand signals, and longitudinal analysis confirms their predictive value in anticipating the conversion to dementia. Additionally, cerebrospinal fluid analyses can identify individuals with amyloid deposits, even in the absence of dementia symptoms. This opens up avenues for identifying individuals at heightened risk of developing dementia and intervening early to reduce amyloid levels, potentially delaying or preventing the onset of AD.

Screening high-risk populations through PET scans and cerebrospinal fluid analyses to detect amyloid signatures could facilitate the identification of individuals with amyloid deposits before significant neural damage occurs. Early intervention targeting amyloid reduction holds promise for slowing down or even preventing the development of Alzheimer’s dementia. Promising results have been observed in the Phase 3 bapineuzumab trial, where PET scans revealed a significant reduction in amyloid signals following 18 months of antibody treatment compared to placebo. Despite challenges like vasogenic edema, other immunotherapy approaches in clinical or preclinical stages offer the potential for reducing amyloid levels before symptom onset, thereby mitigating the cognitive impairments associated with AD. This breakthrough suggests the possibility of preventive measures for high-risk individuals to avert the occurrence of AD.

## 14. Conclusions

Broad-based treatments aim to decimate Aβ42 as it is the key target for causing devastating AD, which includes active immunization and passive immunization. It is an easy mark to target because Aβ42 does not play any physiological role, so it is considered a superfluous fragment like any pathogen. Other than Aβ, the reason for AD is tau protein misfolding and aggregation resulting in neurofibrillary meshes, and α-syn is seen amassed in AD-associated plaque formation. Thus, tau-directed immunotherapies are also in progress and are continuing to remove the leading causes of AD. Anti-tau immunotherapy involving antibodies against misfolded tau proteins and their inclusion mediated by receptors aids in the liberation of AD. The ultimate cure is provided by the exploitation of active immunotherapy, which is still associated with a range of adverse effects as the body’s defense system is unrestrainedly activated. Of the several perilous effects, the major one is amyloid-related imaging abnormality (ARIA). Both therapies offer AD treatment, but only to a limited extent. Likewise, active therapy and passive immunization also impact the body negatively due to the diversified nature of antibodies, but it is still better for their character that antibody management at any time is clogged. Despite potential harms associated with these immunotherapies, there is still hope that the early onset of AD with the administration of an accurately adjusted dose of antibodies against Aβ could also diminish tau and α-syn. Recent studies in mice are indicative that tau epitopes are very toxic and are not only one in number; this is a prospect for cognitive benefits. Depicted antibodies generate such responses during medication, which could be used as biomarkers for AD that help in its early diagnosis. To gain improved and more beneficial effects indicative of future success, combinatorial therapeutics are needed that target more advanced markers of AD for efficient AD treatment.

## Figures and Tables

**Table 2:** Passive immunotherapy clinical trials.

Aβ Passive Immunotherapy Clinical Trials								
Drug	Sponsor	Vaccine Type	Target (Aβ/Tau)	Trial Phase and Status	Immunology	Positive Outcomes	Negative Outcomes	References
Bapineuzumab	Janssen/Pfizer	Humanized monoclonal IgG1 (murine mAb)	N-terminal (Aβ1-5)	Two Phase III studies completed. Terminated, No improvements (NCT00606476, NCT01254773, NCT00996918, NCT00676143, NCT00998764, NCT00667810, NCT00575055, NCT00574132, NCT00916617, NCT00112073)	Fc-mediated activation of microglial phagocytosis and cytokine production. Bind to Aβ monomers, oligomers, and fibrils.	PII: ↓↓CSF t-tau and p-tau while Aβ-40 and 42 remain unchanged. PIII: ↓↓CSF p-tau. In carriers Aβ-42 while in non-carriers Aβ-42, t-tau, and p-tau remain unchanged.	Vasogenic cerebral edema.	[149,159,160,161,162,163]
Solanezumab	Eli Lilly (Indianapolis, IN, USA)	Humanized monoclonal IgG1 (mAb266)	Middle domain (Aβ 16-24) (Aβ monomers)	Two Phase III studies were completed; other phases III tests are ongoing (NCT02760602, NCT01900665, NCT01127633, NCT01148498, NCT02008357, NCT00905372, NCT00749216, NCT00904683, NCT00329082, NCT04623242)	Sequestration of soluble monomers of Aβ thus removes synaptotoxic fragments of Aβ.	PII: ↑↑CSF and Serum Aβ40 and 42 while CSF p-tau and t-tau remain unchanged.	Effects on cognition failed to reach clinical outcomes.	[117,160,164,165,166]
Gantenerumab	Hoffmam- La Rochi (Basel, Switzerland)	Humanized monoclonal IgG1	N terminal and Mid Domain (Aβ 3-12; 18-27)	III Ongoing (NCT02051608, NCT02133937, NCT03236844, NCT03443973, NCT04592341, NCT04339413, NCT02882009, NCT02711423)	Microglia uptake and degradation. Preferentially interacts with fibrillar Aβ, microglial recruitment, and activation.	↓↓Aβ fibrillation.	Vasogenic edema, discontinued after a futility analysis.	[160,167] Scarlet RoAD (NCT01224106; WN25203)
Crenezumab	Genentech (South San Francisco, CA, USA)	Humanized monoclonal IgG4	Mid-domain (oligomers and fibrils)	II/III Ongoing (NCT03491150, NCT03114657, NCT02670083, NCT02427243, NCT01998841, NCT01723826, NCT02353598)	IgG4- Aβ interactions. Selectively targets Aβ oligomers and fibrils.	↑↑Total plasma Aβ level, well-tolerated in mild to moderated AD cases.	Elevated vascular risk (B.P, CVD).	[168,169,170,171]
Ponezumab	Pfizer	Humanized monoclonal IgG2	Aβ 1-40 (C- terminal amino acids) (Plasmatic monomer)	II (Halted) (NCT01125631, NCT00733642, NCT01821118, NCT00455000, NCT01005862, NCT00607308, NCT00722046, NCT00945672)	Peripheral sink.	Safe and well-tolerated. ↑↑plasma Aβ level, ↑↑CSF total Aβ level and free Aβ-42.	Failed to reach primary cognitive endpoints.	[137,172]
Aducanumab	Biogen Idec (Baar, Switzerland)	Humanized monoclonal IgG1	N terminal and Mid Domain (Aβ oligomers and fibrils)	III Ongoing (NCT04241068, NCT02782975, NCT03639987, NCT01677572, NCT01397539, NCT02484547, NCT02477800)	Microglial recruitment and activation.	Brain penetration occurs and ↓↓Aβ stabilization in MMSE and CDR-sb.	Increased ARIA chances, usually in APOE e-carriers.	[173]
BAN2401	BioArtic (Stockholm, Sweden)/Eisai (Nutley, NJ, USA)	Humanized monoclonal IgG1	Protofibrils (≥100 kDa)	II (b) (NCT01230853, NCT02094729, NCT01767311)	Selectively targets soluble Aβ protofibrils.	↓↓CSF-soluble Aβ. Shows a favorable safety profile. protofibrils/oligomers.	No significant adverse effects.	[174,175,176]

↑↑ (increase), ↓↓ (decrease).

**Table 3:** IVIG immunotherapy clinical trials.

IVIG Immunotherapy Clinical Trials								
Drug	Sponsor	Vaccine Type	Target (Aβ/Tau)	Trial Phase and Status	Immunology	Positive Outcomes	Negative Outcomes	References
Octagam IVIG	Octapharma (Charlotte, NC, USA)	Human polyclonal Ab.	Multiple sites on conformational Aβ epitopes	II (Completed, No improvement) (NCT02303093, NCT00504075, NCT02637700, NCT00750867, NCT01859754, NCT01854827, NCT00722475)	Increased Aβ clearance by microglia-mediated phagocytosis.	↓↓Aβ plaques and plasma Aβ-42 level. ↑↑ cognitive functions.	Ischemic stroke and microbleeds.	[182,184]
Gammagard IVIG	Baxter Healthcare (Deerfield, IL, USA)	Human polyclonal Ab	Multiple sites on conformational Aβ epitopes	III (Abandoned) (NCT04153422, NCT00504075, NCT02637700, NCT00750867, NCT01854827, NCT00722475, NCT02042027)	Increased microglial activation and promote Aβ clearance by phagocytosis.	Safe. ↑↑CSF total Aβ, ↑↑plasma Aβ 42 and 40. ↓↓Aβ fibril and oligomer levels.	No improvement.	[97,182] (NCT00818662)
NewGam	Sutter Health (Sacramento, CA, USA)	Human polyclonal Ab	Multiple sites on conformational Aβ epitopes	III Ongoing (NCT02638207, NCT01349790, NCT01012323, NCT01313507, NCT01225276)	Increased Aβ fibril clearance by microglia-mediated phagocytosis. Prevent the formation of soluble Aβ oligomers.	↓↓Aβ fibril and oligomer levels.	-	[185]

↑↑ (increase), ↓↓ (decrease).

## Data Availability

Available on request.

## Abbreviations

Alzheimer’s disease: AD, amyloid β-peptide: Aβ, sporadic Alzheimer’s disease: SAD, familial Alzheimer’s disease: FAD, amyloid precursor protein: APP, central nervous system: CNS, neurofibrillary tangles: NFTs, mitogen-activated protein kinases: MAPK, glial-derived neurotrophic factor: GDNF, triggering receptor expressed on myeloid cells 2: TREM2, complement receptor 1: CR1, beta-amyloid [Abeta]1-42: AN1792, phosphorylated tau: p-tau, general physician: GP, cerebrospinal fluid: CSF, total-tau: t-tau, neurofilament light protein: NFL, functional magnetic resonance imaging: fMRI, magneto-encephalography: MEG, electroencephalography: EEG, single-photon emission computed tomography: SPECT, positron emission tomography: PET, functional near-infrared spectroscopy: fNIRS, Food and Drug Administration: FDA, acetylcholinesterase inhibitors: AChEIs, nonsteroidal anti-inflammatory drugs: NSAIDs, atomic force microscopy: AFM, amyloid related imaging abnormalities: ARIA, intravenous immunoglobulin: IVIG.
